# A novel zero valent metal bismuth for bromate removal: direct and ultraviolet enhanced reduction†

**DOI:** 10.1039/c9ra10391k

**Published:** 2020-01-24

**Authors:** Hong Huang, Guoshuai Liu, Xiuheng Wang

**Affiliations:** State Key Laboratory of Urban Water Resource and Environment, Harbin Institute of Technology Harbin 150090 China xiuheng@hit.edu.cn

## Abstract

Bromate (BrO_3_^−^) is a carcinogenic and genotoxic by-product of the ozone disinfection process. In this study, a new zero-valent metal, bismuth, was used to reduce bromate. Bismuth samples were prepared by a solvothermal method and characterized by powder X-ray diffraction (PXRD), scanning electron microscopy (SEM), and transmission electron microscopy (TEM). The morphology of the bismuth powder was microspheres assembled with dense nanosheets. The kinetics of the direct bromate reduction by bismuth accorded with the pseudo-first-order kinetics model. The rate coefficients of the initial bromate concentration of 1.00 mg L^−1^, 2.50 mg L^−1^, 5.00 mg L^−1^ were identically close to 0.08 min^−1^. For 0.20 mg L^−1^, a reaction rate coefficient near 0.10 min^−1^ was obtained. The reducing products of bromate included bromide ions (Br^−^) and bismuth oxybromides. The bromate removal efficiency was enhanced remarkably in the presence of ultraviolet (UV) light, and the corresponding kinetic coefficient was 4 times higher than that of direct reduction. The mechanism of ultraviolet enhancement was analyzed by diffuse reflectance spectroscopy (DRS), the density functional theory (DFT) calculation, open circuit potential (OCP) analysis, photocurrent measurement and linear sweep voltammetry (LSV). Besides, the influence of dissolved oxygen (DO) on bromate reduction efficiency and the sustainability of the as-prepared sample were investigated. DO inhibited the reduction rate obviously, but showed a slight effect on the formation of bromide ions. In the long-term periodic experiments, the kinetic coefficient decay occurred in both direct (without UV irradiation) and ultraviolet assisted bromate reduction. However, the kinetic coefficient of UV-assisted reduction (0.115 min^−1^) was about 2 times higher than that of the direct reduction in the last cycle of periodic experiments. In conclusion, the novel bromate reduction strategy based on the zero-valent bismuth metal material has been proved efficient and sustainable, which contributes to the development of drinking water treatment technologies.

## Abstract 2

The inert metal bismuth is proved to be effective for the direct reduction of bromate while the reducing process is dramatically promoted under the presence of ultraviolet light, since bismuth is a typical semi-metal.

## Graphical abstract

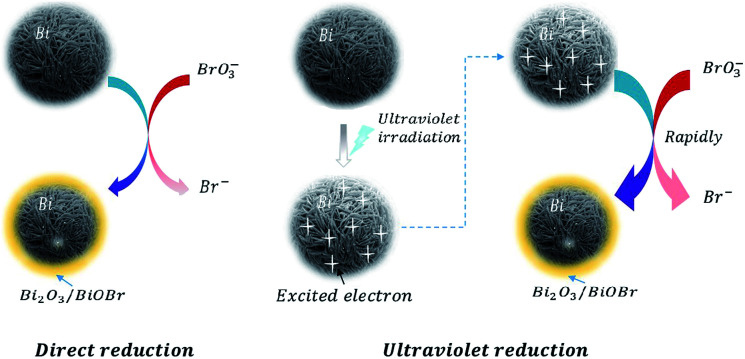

## 1. Introduction

Bromate (BrO_3_^−^) is classified as a Group 2B carcinogen by the World Health Organization (WHO).^1,2^ For potable water, the maximum acceptable concentration of bromate is 10 μg L^−1^, as regulated by the many countries and organizations.^3,4^ The appearance of bromate is attributed to the ozone disinfection process, in which bromide contained in source water is oxidized to bromate *via* direct oxidation by ozone or indirect oxidation by hydroxyl radical.^5–7^ Although over 99% of the global bromine is distributed in seawater and salt lakes, trace bromine in freshwater is still enough to generate excessive bromate.^8^

Strategies of controlling bromate in drinking water can be summed up as three typical processes: pre-disinfection process, disinfection-simultaneous process and post-disinfection process, according to the sequence relation with ozone disinfection process.^9^ The pre-disinfection process focuses on the removal of bromine. The workaround involves ion exchange, ion adsorption and membrane filtration.^10^ The low selectivity for different ions seems problematic for the pre-disinfection process, which disturbs the composition of primary ions in source water and removes some essential trace elements. The disinfection-simultaneous process needs the presence of some special reactants or corresponding scavengers (like ammonia and humic acid).^11,12^ These limitations of the pre-disinfection process and disinfection-simultaneous process make post-disinfection processes an alternative strategy, due to their specificity and efficiency for the decomposition of bromate.

As an essential post-disinfection strategy, zero-valent metal (ZVM) is an easy and highly efficient method of reducing bromate. Several metallic elements are implemented for reducing bromate, such as aluminum and iron.^13,14^ The strong reducing ability (*φ*^θ^(Al^3+^/Al) = −1.667 V) of aluminum makes it theoretically effective for bromate reduction. However, the vigorous reduction ability also induces the rapid surface-oxidation of aluminum in the air, forming a compact oxide layer called the “passivation layer” (or passive oxide layer). The passivation layer baffles the subsequent reaction of bulk aluminum with bromate, thus causing an unexpected low reduction efficiency.^14^ Iron is another crucial metal reducing agent (*φ*^θ^(Fe^2+^/Fe) = −0.43 V) in bromate removal. Zero-valent iron is easily fabricated into the micro-nano level material with large specific surface area and excellent dispersion in water, which is conducive to bromate reduction.^15,16^ However, considering the high sensory, turbidity and chroma requirements of potable water, one critical factor that limiting the application of zero-valent iron on bromate reduction is the trace release of colored trivalent iron into aqueous phase.^17^

Bismuth, located between copper and mercury in metal activity sequence table, lacks sufficient reducing power (*φ*^θ^(Bi^3+^/Bi) = 0.2 V) to react with hydrogen ions and is classified as inert metal. Hitherto, bismuth has rarely been used in the traditional field of zero-valent metal. Nevertheless, bismuth has its particular application potentials for bromate reduction according to the followed reasons. The first point originates from the sufficient thermodynamic for bromate reduction (*φ*((BrO_3_^−^, H^+^ (10^−7^ mol L^−1^))/Br^−^) = 1.008 V). The next one is the low toxicity of bismuth and its compounds. The remarkable innocuity makes bismuth distinctive among the heavy metals, despite its location in the periodic table amid toxic heavy metals.^18^ Many bismuth compounds are even less toxic than table salt and widely used in cosmetic and medicinal chemistry, such as bismuth sub-carbonate, an oral digestive drug (over-the-counter). Moreover, another attractive feature of bismuth is its semimetallic properties,^19^ which means that UV light has the potential to promote its reactivity and may contribute to bromate reduction.^20–22^

As far as we know, there is few relevant investigations reported about the reduction of bromate by bismuth. Herein, we prepared bismuth microspheres assembled with dense nano-sheets by glycol solvothermal method. The as-prepared zero-valent metal was first employed as an effective reducing agent for bromate reduction, and the results demonstrated bismuth is capable of direct reduction of bromate. Furthermore, the ultraviolet (UV) light could enhanced the activity of the bromate reduction significantly. The enhancement mechanism was investigated in depth by experimental characterization and theoretical calculation. Moreover, the influence of dissolved oxygen and the sustainability of the material were investigated. Overall, the feasibility of bismuth for the reduction of bromate could broaden the metal series to inert metals for bromate reduction theoretically, and cause a positive implication on the research of zero-valent metals toward the reduction of bromate.

## 2. Experimental section

### 2.1. Materials

All chemicals were of analytical grade and were used without further purification. Bismuth nitrate pentahydrate, potassium bromate, potassium bromide, ethylene glycol, sodium sulfite, iron powder were purchased from Aladdin Industrial Corporation (Shanghai, P. R. China). TiO_2_ (P25) was obtained from Evonik Degussa Specialty Chemicals Co. Ltd. Deionized (DI) water was produced by an ultrapure water system. Absolute ethanol was purchased from Sinopharm (China). The deoxidization deionized water (DDI) and deoxidization absolute ethanol (DAE) were prepared by heating and ultrasound. The prepared DDI and DAE were stored in iodimetry bottles with liquid seal and used within no more than 3 hours. Subsequent experiments were carried out using the deoxidization deionized water or deoxidization absolute ethanol if no special notice was given.

### 2.2. Synthesis of bismuth

In a typical synthesis process, 0.61 g bismuth nitrate pentahydrate were added into 50 mL ethylene glycol under continuous stirring until the solution became transparent. Then the solution was transferred into a 100 mL Teflon-lined stainless steel autoclave. The condition of the solvent-thermal process was 185 °C for 24 hours. After the stainless steel autoclave cooled to room temperature, the precipitate was centrifuged for solid–liquid separation. The synthesized sample was successively washed by deoxidization deionized water and deoxidization absolute ethanol and then dried at 60 °C in a vacuum for 6 hours. The final obtained product was preserved in a nitrogen atmosphere together with bagged iron powder.

### 2.3. Characterization

The crystal structures of sample was analyzed by powder X-ray diffraction (PXRD), conducted on an X-ray diffractometer (Bruke D8 Adv., Germany) with Cu Kα radiation at 40 kV and 30 mA, ranged from 10° to 70° at the counting time of 10 s and scanning step of 0.02°. Ultraviolet-visible (UV-Vis) diffuse reflectance spectra was measured on a UV-Vis spectrophotometer (UV-2550, Shimadzu, Japan). The morphologies and microstructures of samples were characterized by scanning electron microscopy (SEM, JEOL JSM-6700F, Japan) and transmission electron microscopy (TEM, F-30ST, US). The specific surface areas of the samples were analyzed by nitrogen adsorption in a Tristar 3000 nitrogen adsorption apparatus.

### 2.4. Reduction of bromate

In a typical reduction experiment, 0.01 g prepared sample was homogeneously dispersed into 50 mL of bromate solution in a quartz test tube under continuous stirring. For a given time interval, 2.5 mL of solution was taken out and then centrifuged by 3000 rpm min^−1^ for solid–liquid separation. The obtained supernatant (2.0 mL) was filtrated by a 0.22 μm membrane filter and further analyzed. The precipitate-part was carefully washed out with no more than 2 mL DDI and then transfer back to the reactor in recycling reduction experiments. For ultraviolet-assisted (UV-assisted) reduction, the experiments were performed in 50 mL quartz tubes rotating around a 500-W high pressure mercury lamp (CEL-LAB500, China). Spectrum of high pressure mercury lamp was provided in Fig. S1.† The intensity was 50 mW cm^−2^ measured by thermopile (Newport 818P-010-12). In order to eliminate the effect of oxygen in air on reduction reaction, the reactor was keeping continuous nitrogen-aeration if no special notice was given.

### 2.5. Element analysis

The inductively coupled plasma atomic emission spectrometer meter (ICP-AES, Optima8300, US) was used for bismuth content analysis. Bromate and bromide ion in the liquid phase were analyzed *via* the ion chromatography system (ICS-1600, US). Free available bromine (consists of dissolved bromine, hypobromous acid and hypobromite) was detected by the method of neutral *N*,*N*-diethyl-*p*-phenylenediamine (DPD) spectrophotometry.^23^ X-ray photoelectron spectroscopy (XPS, Kratos Axis Ultra DLD, Japan) was adapted to analyze the trace bromine in the surface of reacted samples.

### 2.6. Theoretical calculations

A periodic density functional theory (DFT) package of CASTEP codes was used for theoretical calculations in the present work. CASTEP was widely adopted in the theoretical calculation of semiconductor properties. The functional was local-density approximation (GGA-PBE), tolerance was 1.0 × 10^−5^, and the core treatment was all-electron. The electronic structure and the optical properties were calculated basing on the optimized molecular structure.

### 2.7. Photo-electrochemical measurements

A conventional three-electrode electrochemical cell was adapted for electrochemical and photo-electrochemical measurements. The working electrode was prepared by drop-casting method on indium tin oxides (ITO) glass (1 cm × 1 cm). The same size platinum foil acted as a counter electrode, and a saturated calomel electrode (SCE) served as a reference electrode. All the electrodes were immersed in a sodium perchlorate solution (0.1 M). The collection of electric signals was taken on a CHI660C workstation. For photo-electrochemical measurement, the irradiated condition was the same as the reduction experiment.

## 3. Results and discussion

### 3.1. Crystal structure and morphology

**Fig. 1:**
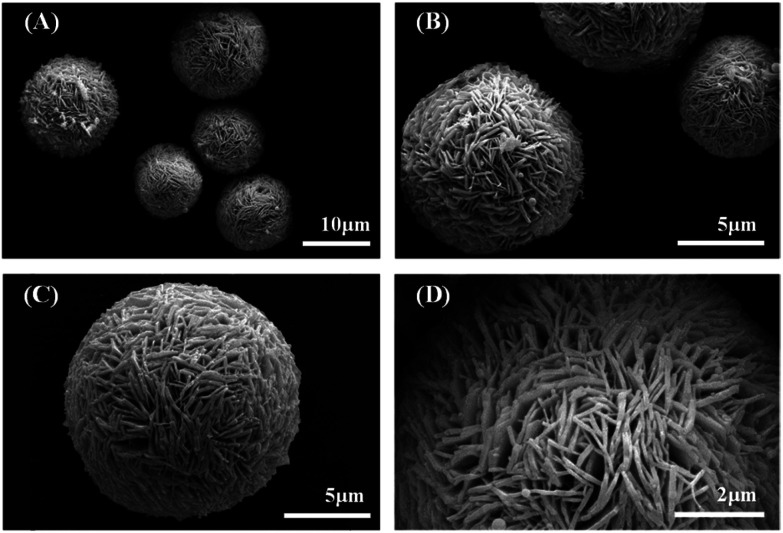
(A–D) SEM images of the synthesized bismuth microspheres.

The PXRD pattern of the synthesized powder is shown in Fig. S2.† The structure has a good agreement with the pure rhombohedral Bi (JCPDS no. 44-1246, space group *R*3*m*, lattice constants *a* = *b* = 4.567(6) Å and *c* = 11.91(1) Å), no impurities were observed. The SEM images show that the synthesized Bi has a hierarchical structure of microspheres, with diameters of 10.0–15.0 μm, assembled with dense nanosheets. For nanosheets, the diameters of 1.0–2.5 μm is far greater than the thickness of 0.1–0.2 μm (in Fig. 1).

**Fig. 2:**
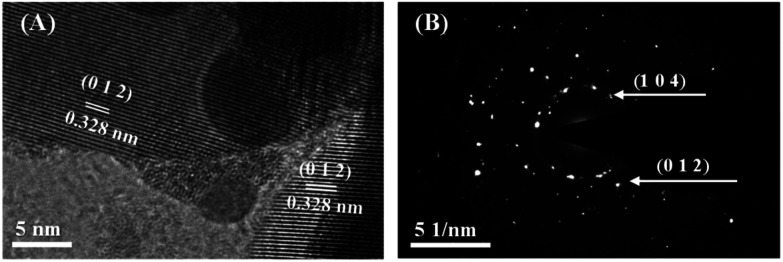
(A) TEM image of the synthesized bismuth nanosheets. (B) SAED pattern of the synthesized bismuth nanosheets.

The HRTEM observation (Fig. 2A) shows a lattice spacing of 0.328 nm for the synthesized nanosheets, indicating the exposed facets is (0 1 2). The selected area electron diffraction (SAED) pattern (Fig. 2B) demonstrated that the synthesized bismuth powder had an excellent crystallinity. Continuous sharp circles with *d*-spacing of 0.328 nm and 0.237 nm could be clearly identified, which corresponded to (0 1 2) and (1 0 4) lattice planes, respectively.^24^

The BET results showed the specific surface area is about 10.0 m^2^ g^−1^ (95% confidence intervals: 8.9 m^2^ g^−1^ to 11.1 m^2^ g^−1^), and the pore-size distribution of mesoporous is around 10.5 nm. The BET specific surface area is comparable with the reported zero-valent iron (5.2 m^2^ g^−1^),^25^ which indicates the prepared sample maintained sufficient active sites for reducing bromate.

### 3.2. Bromate reduction performances of Bi microspheres

For evaluating the reduction performance of the synthesized Bi powder, a serious of experiments were carried out for comparative observation. The direct reduction experiment with gradient initial bromate concentration was carried out without UV irradiation.

**Fig. 3:**
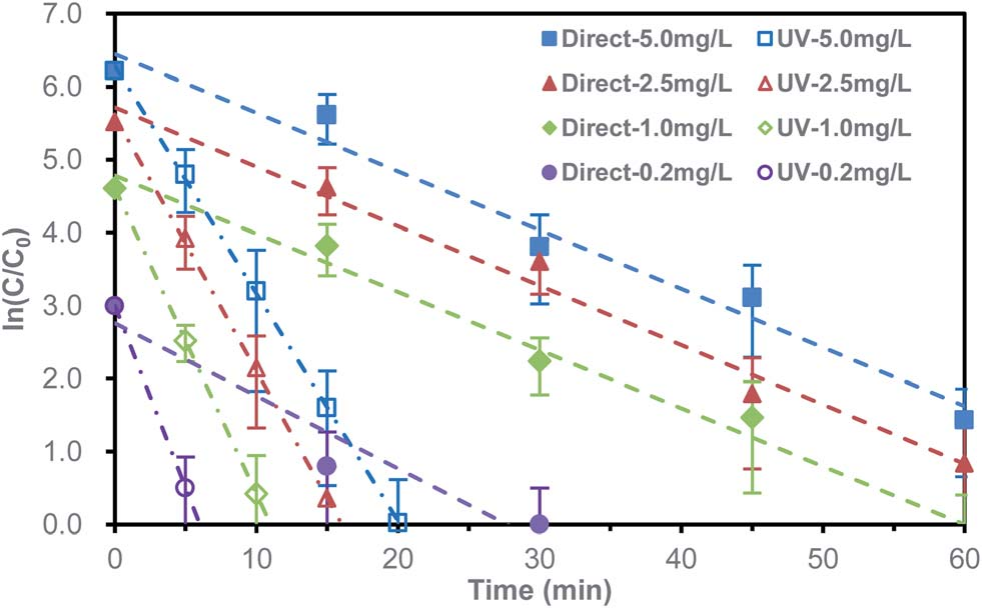
Bromate reduction in logarithmic concentration ratio form for direct and UV-assisted reduction at different initial bromate concentration.

The direct bromate reduction acted as a pseudo-first-order kinetic behavior. The rate coefficient (*k*) was obtained by liner-fitting of reaction time (*t*) and the logarithm of corresponding concentration ratio (ln(*C*/*C*_0_)). Herein, *C*_0_ was specially defined as 10 μg L^−1^, representing for the maximum acceptable bromate concentration,^3,4^ rather than the initial concentration. This data-processing method detailedly represented the variation of low concentration, as in Fig. 3 and Table S1.† When the initial concentration was 0.2 mg L^−1^, the bromate concentration decreased to less than 10 μg L^−1^ in 30 minutes. With the increase of initial bromate concentration, the time needed for bromate removal to below 10 μg L^−1^ increased correspondingly. For 1.0 mg L^−1^, it took about 60 minutes, while for 2.5 mg L^−1^ and 5.0 mg L^−1^, the removal efficiency was not reached to target concentration in the 60 minutes reaction period. The rate coefficients of the initial bromate concentration of 1.00 mg L^−1^, 2.50 mg L^−1^, 5.00 mg L^−1^ was respective 0.080 ± 0.018 min^−1^, 0.081 ± 0.019 min^−1^ and 0.080 ± 0.023 min^−1^. For 0.20 mg L^−1^, a faster reaction rate coefficient of 0.100 ± 0.046 min^−1^ was obtained. The faster reaction rate coefficient of initial 0.20 mg L^−1^ may related to the adsorption of surface oxides formed during sample preparation.

For the bromate reducing process, bromide ion is an ideal reducing product. Dissolved bromine, hypobromous acid and hypobromite (collectively called “free available bromine”) are not ideal reducing product, as they are still oxidizing and may react with organic substances in the human body. Free available bromine was not detected (<0.01 mg L^−1^) during the reaction time. Bromide ions appeared as the only reducing products in the liquid phase. However, the decrease of the total bromine (the sum of bromate, free available bromine and bromide ion) in solution was remarkable, as in Fig. S3.† The proportion of the final total bromine in liquid to initial bromine was 0.0%, 29.2%, 48.6% and 45.1%, respectively for the initial bromate of 0.20 mg L^−1^, 1.00 mg L^−1^, 2.50 mg L^−1^ and 5.00 mg L^−1^.

In order to interpret the bromine transformation, XPS analysis was taken on the reacted Bi microspheres in the experiment of initial 5.00 mg L^−1^. The XPS result in Fig. S4B† showed a signal peak near 68.0 eV. By comparative analysis,^26^ the signal peak ascertained the presence of bromide on the surface of the reacted bismuth microspheres. The reason why bromide appeared in the solid surface was not only the adsorption of the double electric layer of materials but also the insolubility of bismuth oxyhalide.^27^ The above analysis demonstrated the synthesized Bi sample was able for direct bromate reduction.

Unlike most metals, the semimetallic properties of bismuth made it possible to introduce UV for improving reactivity.^20–22^ The ultraviolet-assisted bromate reduction by bismuth microspheres was shown in Fig. 3 and Table S2.† Comparing to the former direct reduction, introducing UV produced an exciting situation, which significantly promoted the reduction of bromate. At the initial concentration of 0.2 mg L^−1^, the bromate concentration decreased to less than 10 μg L^−1^ in about 6.5 min. When the initial bromate concentration was 1.0 mg L^−1^, 2.5 mg L^−1^ and 5.0 mg L^−1^, it took 11 min, 16.5 min and 20 min, respectively. The rate coefficient (*k*) assisted by ultraviolet radiation were 0.499 ± 0.042 min^−1^, 0.419 ± 0.031 min^−1^, 0.349 ± 0.013 min^−1^ and 0.312 ± 0.013 min^−1^, respective for the initial bromate concentration of 0.20 mg L^−1^, 1.00 mg L^−1^, 2.50 mg L^−1^ and 5.00 mg L^−1^. The corresponding coefficient was 5.0, 5.2, 4.4 and 4.0 times as fast as that of direct reduction (arrange from low initial concentration to high initial concentration). The observed phenomenon indicated the UV light dramatically enhanced the bromate removal on efficiency.

The proportion of the final total bromine in liquid to initial bromine was 16.2%, 18.1%, 25.6% and 66.5%, respectively for the initial bromate of 0.20 mg L^−1^, 1.00 mg L^−1^, 2.50 mg L^−1^ and 5.00 mg L^−1^. Only bromide ions appeared in the solution, and no free available bromine was detected. Fig. S4C† showed the existence of bromide on the solid surface by XPS analysis. The result was in line with the aforementioned results of the direct reduction, which exhibited two transformation pathways for bromine transformation, *i.e.*, the transformation towards bromide ions in the aqueous solution and the formation of bismuth oxybromides on the solid surface of bismuth microspheres.

For drinking water, the concentration of metal ions in the water is a crucial indicator. Excessive intake of metal ions will cause toxic effects, such as iron^28^ and aluminum.^29^ Excessive bismuth intake lead to accumulation in tissues and causes problems in kidneys, liver and circulatory systems.^30^ The residual bismuth in the treated water should be regulated. The results of residual bismuth in the liquid phase after reaction for various initial bromate concentrations were presented in Table S6.† The concentration of residual bismuth kept at a low level of 10–20 μg L^−1^. By comparable experiments (in Table S7†), the effect of mechanical agitation on the concentration of residual bismuth was severe. Mechanically agitation caused mechanical abrasion of bismuth microspheres, which consisted of the collision between particles, the collision between particles and vessel wall, and the falling-off and dissolution of particles surface substance. The residual bismuth caused by mechanical abrasion could be reduced by adopting a suitable reactor, which reduced particle collision and ensured mass transfer efficiency.^31,32^ For terminal controlling of residual bismuth, filtration was practicable. In this paper, simple natural clinoptilolite (60 mesh) filtration (0.5 m h^−1^, 15 cm) was used, and bismuth residue was not detected (<0.3 μg L^−1^).

Based on the above analysis, the synthesized Bi sample was able for bromate reduction and achieved acceptable removal efficiency. More importantly, ultraviolet radiation significantly accelerated the reduction activity of bismuth to bromate.

### 3.3. Mechanism of ultraviolet promotion

**Fig. 4:**
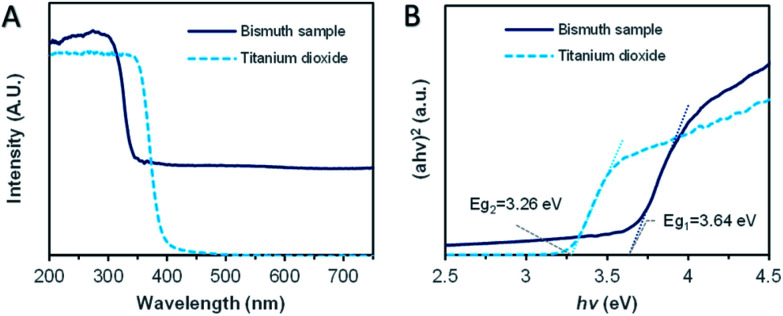
The ultraviolet-visible (UV-Vis) diffuse reflectance absorption spectrum of Bi sample and TiO_2_ (P25). (A) The UV-Vis diffuse reflectance spectrum. (B) The absorption edge analysis.

For understanding the promotion mechanism of ultraviolet radiation, the absorption characteristics of bismuth microspheres were analyzed. The UV-Vis diffuse reflectance spectroscopy of synthesized Bi microspheres and reference typical semiconductor sample TiO_2_ (P25) were displayed in Fig. 4A. In the ultraviolet band, the absorption band edge located around 330 nm. The corresponding photon energy, as calculated, was 3.64 eV, as in Fig. 4B, which was lower than the workfunction of bismuth (4.20 eV).^33^ In the visible band, bismuth samples showed non-zero stationary absorption, the specific reason we will discuss below. The absorption characteristics of bismuth were different from that of typical semiconductors. For TiO_2_, the absorption edge was located at 383 nm, the corresponding bandgap is 3.26 eV, but there was no absorption for the photo with a wavelength of more than 400 nm.

**Fig. 5:**
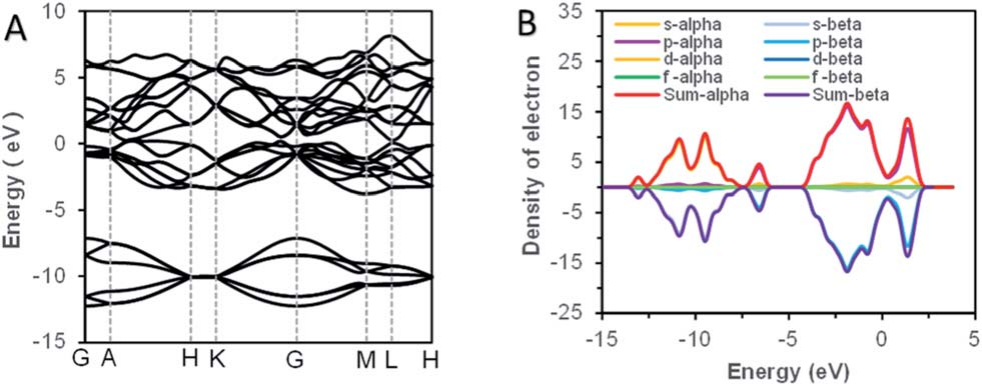
The result of band structure theoretical calculations. (A) The energy band structure of Bi. (B) The density state of Bi.

As we know, the optical absorption properties of materials are mainly the result of the electron transition between electronic orbits. According to the previous analysis by SAED, the bismuth samples were highly crystalline. For crystal solid materials, the energy band structure was widely adopted to analyze the electronic orbital structure, and the theoretical calculation of first-principles was used to explain the energy band structures.^34^ The theoretical calculation results of bismuth were shown in Fig. 5. The characteristic ultraviolet absorption of bismuth mainly caused by the band–band electron transition from the deep valence band (around −2.5 eV below the Fermi level) to the bottom of the conduction band.^21^ For visible light, the absorption was ascribed to the partial overlaps of the valence band and conduction band. Usually, a block bismuth was highly absorbing and highly reflective materials, with silvery-white appearance. However, as the thickness of the synthesized bismuth nanosheet was lower than the visible wavelength (400–720 nm), the reflection of the powder to visible light was significantly weakened. The size effect made the synthetic bismuth powder absorb but weakly reflect visible light, which caused the non-zero stationary absorption in the visible band.

Although bismuth absorbed both ultraviolet and visible light, the contrast experiment showed that the enhancement effect of UV was much stronger than that of visible light (in Fig. S5†). Under UV light, the photolysis ratio of bromate was about 4% in 60 minutes, which excluded the decrease of bromate caused by ultraviolet photolysis. Under visible light irradiation (Xenon lamp, 500 W), the kinetic rate of bromate degradation was close to direct reduction. It was evidently that bismuth got better reduction performance under the UV condition. The difference absorption intensity in UV band and Vis band was an important and basic influence factor for different enhancement effects. However, it is reasonable to believe other factors also play important roles in the differences, such as the influence of surface modification, crystal defects and the transition state of excited electron.^35–37^

The results of UV-Vis diffuse reflectance spectroscopy proved the as-synthesized bismuth powder was able to absorb ultraviolet light and produce photoexcited electrons with specific reduction potential. For the electron excitation process, the specific reduction potential of the excited electrons was more negative than the potential corresponding to the Fermi level.^38^ The Fermi level of bismuth is −4.2 eV,^33^ and the corresponding redox potential is −0.3 V. Under neutral conditions, the redox potential of bromate is 1.008 V. Therefore, the ultraviolet-excited electrons offer sufficient thermodynamic impetus for bromate reduction (Δ*E* ≫ 0.4 V).

**Fig. 6:**
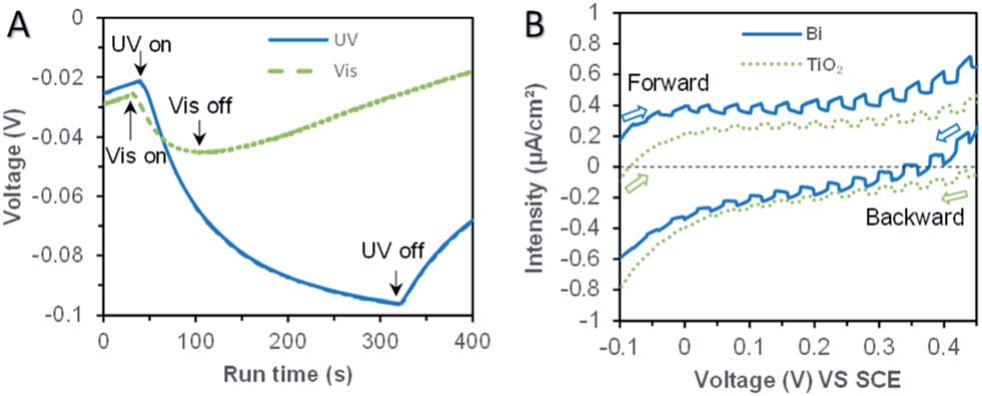
The result of photo-electrochemical measurements. (A) Change of open circuit voltage under UV and visible irradiation for pure bismuth sample. (B) LSV under periodic ultraviolet irradiation of bismuth sample and reference TiO_2_ (P25).

The thermodynamic reduction potential change was intuitive through the open-circuit potential (OCP) changes of the bismuth microspheres electrode (in Fig. 6A). The OCP value was more negative under the UV irradiation, this indicated the bismuth was favorable for bromate reduction under the assistance of UV irradiation. This phenomenon was direct evidence responsible for the enhancement for bromate reduction in thermodynamic viewpoint.

The above analysis demonstrated the thermodynamic feasibility of the bromate reduction by bismuth photoexcited electrons, but the kinetic feasibility (focus on the possibility of transmitting electrons outside) was another parameter and dominated the actual reduction rate of bromate. Photocurrent measurements under UV conditions were used to characterize the kinetic properties of photo-excited electrons in bismuth samples. Fig. S6† displayed the photocurrent generation under regular intervals of ultraviolet light. Bismuth powder and reference TiO_2_ both produced photocurrent. The photocurrent intensity of bismuth powder was the higher, about 4 × 10^−7^ A cm^−2^, and the photocurrent intensity of TiO_2_ was 8 × 10^−8^ A cm^−2^.

**Fig. 7:**
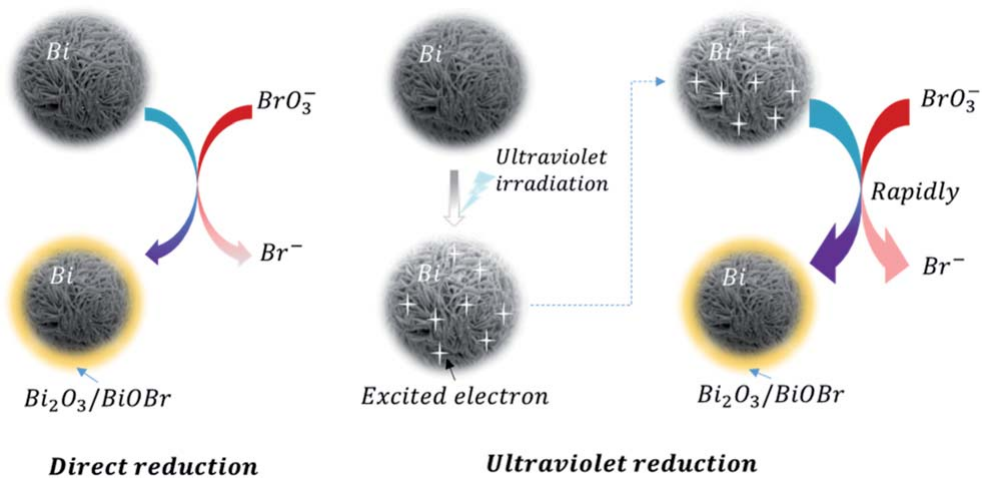
The direct bromate reduction and ultraviolet enhanced bromate reduction by bismuth.

Furthermore, linear sweep voltammetry (LSV) under periodic ultraviolet irradiation was investigated. The results of linear sweep voltammetry showed that bismuth and TiO_2_ produced obvious photocurrent in a wide potential range, which met the need of bromate reduction, as shown in Fig. 6B. Thus, the OCP measurement and photocurrent analysis demonstrated the prepared bismuth sample owned robust thermodynamic and kinetic feasibility of bromate removal, which responsible for the high bromate removal efficiency and selectivity in the presence of UV light. According to the systemic experimental investigation and theoretical evidence, a manifest mechanism scheme was proposed as shown in Fig. 7.

### 3.4. The influence of dissolved oxygen and the sustainability of bismuth microspheres

For the influencing factors of bromate reduction (direct reduction by metal and light-assisted reduction), many studies have been carried out on bromate concentration, reductant/catalyst concentration, hydrogen ion (pH value) and other anions/cations.^39–42^ However, given the particular bromate reduction system by bismuth, two typical and crucial influencing factors were chosen for discussion in this paper. The first is the residual dissolved oxygen. Ozone disinfection not only produces by-product bromate but also dissolved oxygen.^43^ The residual dissolved oxygen is highly likely to compete with bromate in subsequent reduction reactions. The second is the sustainability of the catalyst. The surface of bismuth microspheres was continuously oxidized to bismuth oxide or bismuth oxybromide in direct or ultraviolet-assisted reduction and may gradually lose its direct-reducing ability.

Schemes of periodic contrast experiments were designed to evaluate sustainability. Nitrogen-aeration/air-aeration was used to control the dissolved oxygen level in contrast experiments. At the end of each cycle, the initial bromate concentration (1.0 mg L^−1^) of the reactor was initialized by dropping high concentration bromate solution (0.5 mL, 100 mg L^−1^) and supplying water to the given scale of 50 mL.

**Fig. 8:**
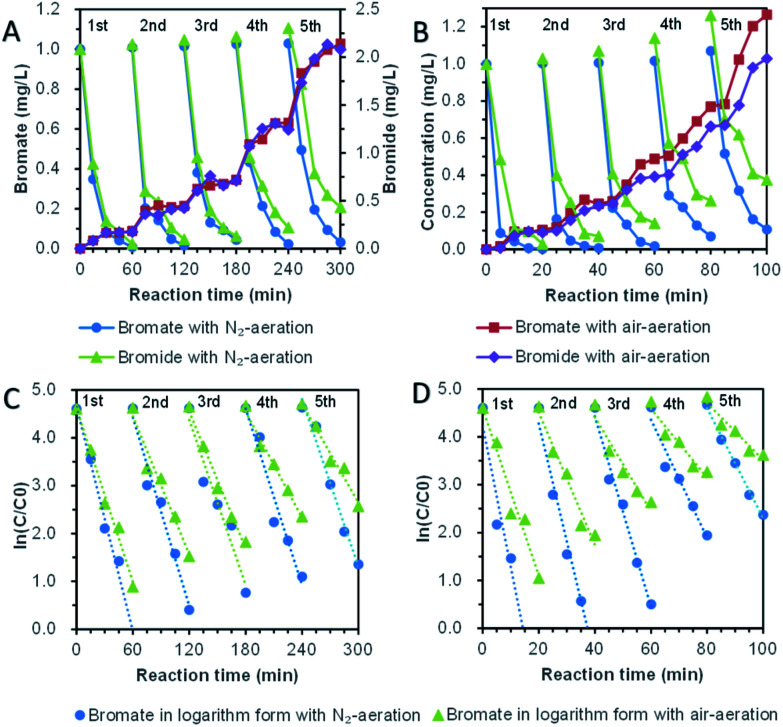
Periodic contrast experiments for direct bromate reduction with nitrogen-aeration/air-aeration. (A) Periodic variation for bromate and bromide ions for direct bromate reduction. (B) Periodic variation for bromate and bromide ions for UV-assisted reduction. (C) Pseudo first order dynamics fitting for periodic direct bromate reduction. (D) Pseudo first order dynamics fitting for periodic UV-assisted bromate reduction.

Five repeated cycle-experiments of direct bromate reduction by bismuth were shown in Fig. 8A and C. Although the pseudo-first-order dynamic model was distorted when it was applied in multi-period operation, it was still adopted basing on the advantages of manifest expression. For five cycles with nitrogen-aeration, the kinetic coefficient decreased from the highest 0.078 ± 0.015 min^−1^ to the lowest 0.058 ± 0.016 min^−1^, with a reduced ratio of 26.4%. In the air-aeration group, the kinetic coefficient decreased from the highest 0.060 ± 0.012 min^−1^ to the lowest 0.034 ± 0.011 min^−1^, with a reduced ratio of 43.2%. The average kinetic coefficient of bromate removal was 0.064 min^−1^ with nitrogen-aeration and 0.045 min^−1^ with air-aeration. It was obvious that dissolved oxygen reduces the removal rate of bromate. Whether dissolved oxygen was controlled or not, the kinetics of direct reduction of bromate showed significant attenuation, and the attenuation degree of air-aeration was higher than nitrogen-aeration. The concentration of bromide ion accumulated, and the ratio of bromide ion to the final product increased with the reaction time.

Five repeated cycle-experiments (in Fig. 8B and D) of bromate reduction by bismuth under UV light ultraviolet were carried out. For nitrogen-aeration group, pseudo-first-order kinetics were 0.294 ± 0.090 min^−1^, 0.246 ± 0.062 min^−1^, 0.199 ± 0.048 min^−1^, 0.123 ± 0.053 min^−1^ and 0.115 ± 0.018 min^−1^. The kinetic coefficients of the fifth cycle decreased to 39% of the first cycle. Under the condition of air-aeration, the kinetic coefficients for the five-cycle experiments were 0.174 ± 0.066 min^−1^, 0.138 ± 0.047 min^−1^, 0.098 ± 0.050 min^−1^, 0.072 ± 0.029 min^−1^ and 0.059 ± 0.29 min^−1^. The coefficient of the last period is 34% of that of the first period. Oxygen reduced the reduction rate of bromate, and the reducing ability of bismuth decreased with the increase of cycle. However, the kinetic coefficients of the fifth cycle under UV irradiation were still quite fast compared with the previous reports.^44–47^ The phenomenon probably was because bismuth oxide and bismuth oxybromides are semiconductors,^48–51^ which were able to degrade bromate continuously under UV light. The phenomenon provided another important reason for the introduction of UV irradiation, which was helpful for building a long-term bromate reduction system.

## 4. Conclusions

In this paper, a novel zero-valent bismuth metal was used for bromate reduction. Crystalline rhombohedral bismuth with the morphology of microspheres assembled with nanosheets was synthesized by the glycol solvothermal method. The as-prepared bismuth microspheres were firstly employed in bromate reduction. Specifically, the direct reduction process accorded with the pseudo-first-order kinetic model, and the corresponding kinetic coefficients were below the value of 0.10 min^−1^. The products for bromate reduction contained bromide ions and bismuth oxybromides. According to the semi-metallic properties of bismuth metal, UV light was introduced to enhance the reducing ability of bismuth metal, and the corresponding kinetic coefficient increased more than 400% compared to the direct reduction. The OCP measurement and photocurrent analysis demonstrated that the excited electrons in bismuth sample owned robust thermodynamic and kinetic feasibility of bromate removal, which was responsible for the high bromate removal efficiency and selectivity in the presence of UV light. Besides, the UV-assisted bromate reduction system by bismuth overcomed the disadvantages of DO inhibition, which exhibited slight kinetic decay and excellent sustainability. In brief, the remarkable reduction enhancement by ultraviolet radiation provided an essential breakthrough for improving the reducing ability of semi-metallic elements. This innovative technology is potentially applicable for controlling the excessive bromate in drinking water, and inspires the development of zero-valent metal reduction technology from active metal to inert metal.

## Conflicts of interest

There are no conflicts to declare.

## Supplementary Material

RA-010-C9RA10391K-s001
