# Supplementary material for: A novel zero valent metal bismuth for bromate removal: direct and ultraviolet enhanced reduction
Source: RSC Adv. 2020 Jan 24;10(7):4148–55. doi: 10.1039/c9ra10391k (PMC9049030; doi:10.1039/c9ra10391k)
Supplement: RA-010-C9RA10391K-s001 [file RA-010-C9RA10391K-s001.pdf]

Electronic Supplementary Material (ESI) for RSC Advances.
This journal is © The Royal Society of Chemistry 2020

Supporting Information

A Novel Zero Valent Metal Bismuth for Bromate Removal: Direct and Ultraviolet Enhanced Reduction

Hong Huang[a], Guoshuai Liu[a], Xiuheng Wang[a]*

[a] State Key Laboratory of Urban Water Resource and Environment, School of Environment, Harbin Institute of Technology, Harbin 150090, P. R. China.

Corresponding author:

* Xiuheng Wang

P. O. Box 2603#, No. 73, Huanghe Road, Nangang District, Harbin, 150090, China.

Tel.: +86–451–86283008; Fax: +86–451–86282110

E–mail: xiuheng@hit.edu.cn

Summary

Number of Pages: Page S1–Page S8

Number of Figures: Figure S1–Figure S6

Number of Tables: Table S1–Table S7

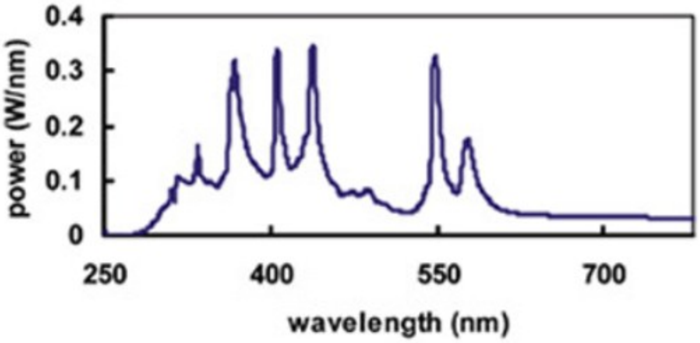

Figure S1 Spectrum of high ressure mercury lamp.

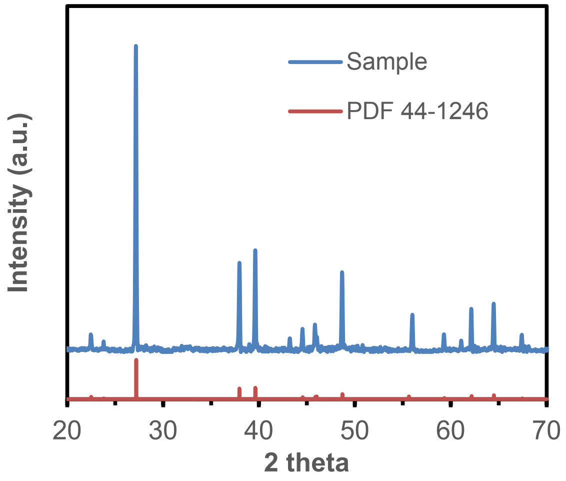

Figure S2 The PXRD pattern of the synthesized sample and the pure rhombohedral Bi (JCPDS no. 44-1246).

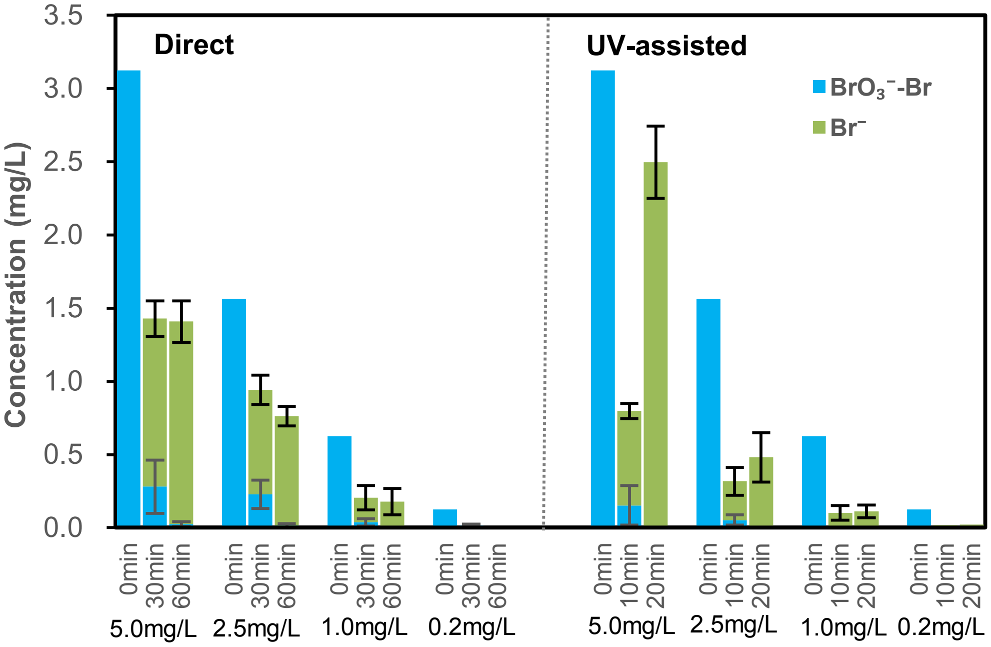

Figure S3 Bromine thansform in liquid phase at different initial bromate concerntration for direct and UV-assisted reduction.

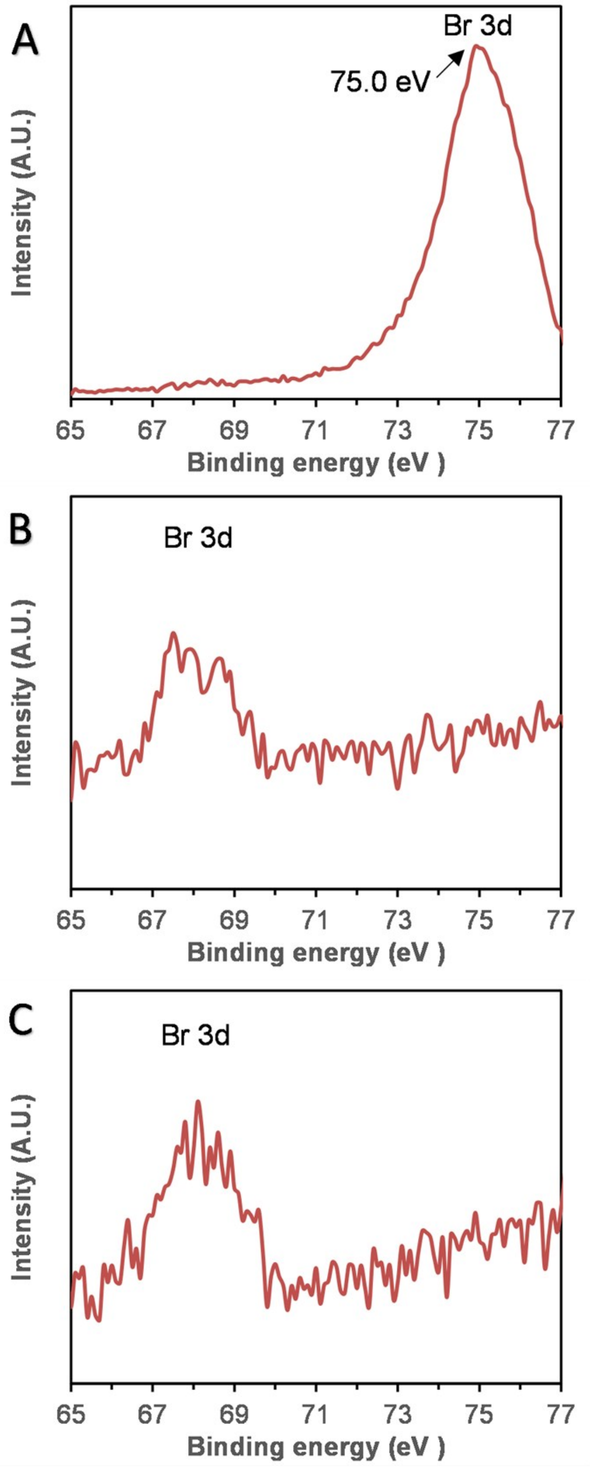

Figure S4 The result of X-ray photoelectron spectroscopy. (A) For pure bromate. (B) For the reacted Bi sample in direct reduction. (C) For the reacted Bi sample in ultraviolet-assisted reduction.

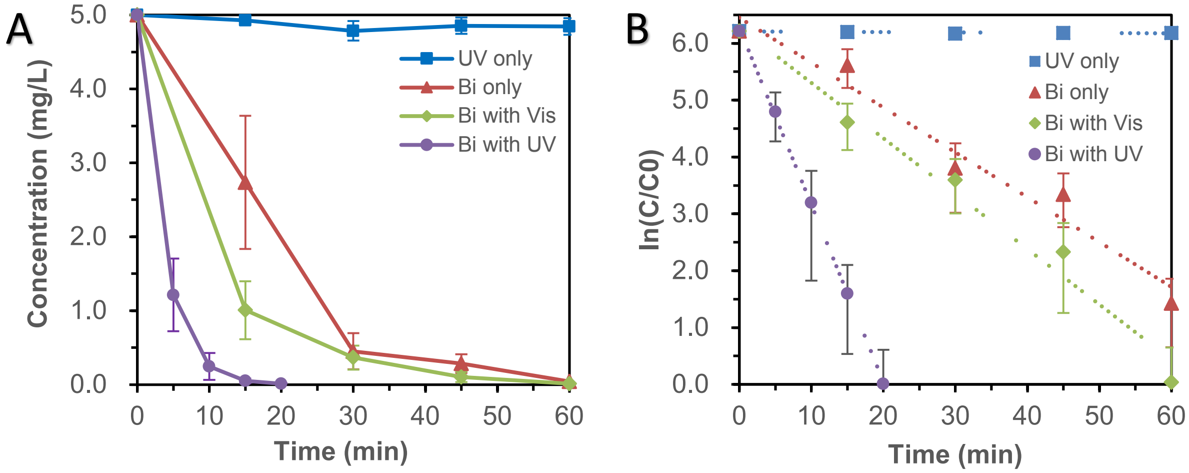

Figure S5 Comparative experiment of bromate degradation. (Four scenarios: ultraviolet light only (UV only), bismuth only (Bi only), bismuth with visible light (Bi with Vis), and bismuth with ultraviolet light (Bi with UV)). (A) Change of bromate concentration. (B) Change of bromate concentration in logarithmic concentration ratio form.

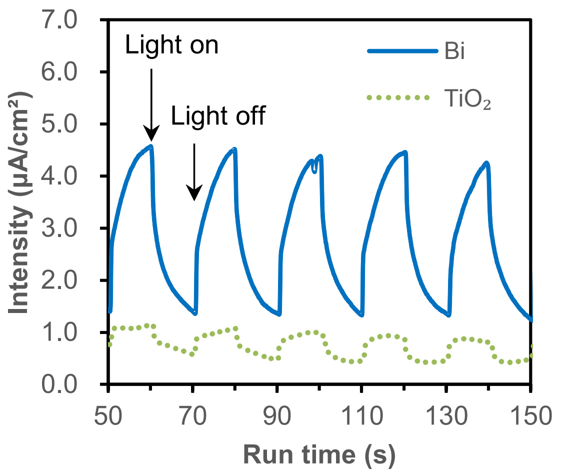

Figure S6 Photocurrent measurements of bismuth sample and reference TiO_2 (P25).

Table Lists

Table S1. Pseudo-first-order kinetic coefficient of direction reduction.

Initial concentration of bromate	kinetic coefficient (min^{-1})	95% confidence intervals (min^{-1})
0.2 mg/L	0.100	0.054-0.146
1.0 mg/L	0.080	0.062-0.098
2.5 mg/L	0.081	0.063-0.100
5.0 mg/L	0.080	0.057-0.104

Table S2. Pseudo-first-order kinetic coefficient of ultraviolet-assisted reduction.

Initial concentration of bromate	kinetic coefficient (min^{-1})	95% confidence intervals (min^{-1})
0.2 mg/L	0.499	0.457-0.541
1.0 mg/L	0.419	0.388-0.450
2.5 mg/L	0.345	0.336-0.363
5.0 mg/L	0.309	0.299-0.325

Table S3. Pseudo-first-order kinetic coefficient in comparative experiment of bromate degradation.

Experimental condition	kinetic coefficient (min^{-1})	95% confidence intervals (min^{-1})
Ultraviolet light only	0.0005	-0.0004-0.0014
Bismuth only	0.078	0.062-0.098
Bismuth with visible light	0.098	0.071-0.124
Bismuth with ultraviolet light	0.312	0.299-0.325

Table S4. Pseudo-first-order kinetic coefficient of periodic contrast experiments in direct reduction.

Experimental condition	kinetic coefficient (min^{-1})	95% confidence intervals (min^{-1})
1st cycle with nitrogen-aeration	0.078	0.063-0.093
2nd cycle with nitrogen-aeration	0.066	0.045-0.086
3rd cycle with nitrogen-aeration	0.061	0.032-0.083
4th cycle with nitrogen-aeration	0.058	0.036-0.086
5th cycle with nitrogen-aeration	0.058	0.042-0.074
1st cycle with oxygen-aeration	0.060	0.048-0.072
2nd cycle with oxygen -aeration	0.048	0.031-0.065
3rd cycle with oxygen -aeration	0.048	0.038-0.057
4th cycle with oxygen -aeration	0.037	0.029-0.045
5th cycle with oxygen -aeration	0.034	0.023-0.045

Table S5. Pseudo-first-order kinetic coefficient of periodic contrast experiments in ultraviolet-assisted system.

Experimental condition	kinetic coefficient (min^{-1})	95% confidence intervals (min^{-1})
1st cycle with nitrogen-aeration	0.294	0.204-0.383
2nd cycle with nitrogen-aeration	0.246	0.184-0.309
3rd cycle with nitrogen-aeration	0.199	0.151-0.247
4th cycle with nitrogen-aeration	0.124	0.070-0.177
5th cycle with nitrogen-aeration	0.115	0.097-0.133
1st cycle with oxygen-aeration	0.174	0.109-0.240
2nd cycle with oxygen -aeration	0.138	0.091-0.185
3rd cycle with oxygen -aeration	0.098	0.048-0.148
4th cycle with oxygen -aeration	0.072	0.038-0.106
5th cycle with oxygen -aeration	0.059	0.030-0.089

Table S6. The residual bismuth in liquid phase after the direct/UV-assisted reduction reaction.

Initial concentration of bromate	Residual bismuth (μg/L)
0.2 mg/L	15.6 ± 1.8
1.0 mg/L	11.7 ± 1.6
2.5 mg/L	15.2 ± 0.7
5.0 mg/L	23.5 ± 1.1

Table S7. The residual bismuth in liquid phase after the direct/UV-assisted reduction reaction.

Initial concentration of bromate	Residual bismuth (μg/L)
No bromate addtion, with mechanically agitated, without UV, with N2- aeration	10.6 ± 1.3
No bromate addtion, without mechanical agitation, without UV, with N2- aeration	0.6 ± 0.2
